# The human factor between airborne pollen concentrations and COVID-19 disease dynamics

**DOI:** 10.1073/pnas.2107239118

**Published:** 2021-08-16

**Authors:** Cornelia Betsch, Philipp Sprengholz

**Affiliations:** ^a^Communication Science, University of Erfurt, 99089 Erfurt, Germany

Damialis et al. (1) report that higher airborne pollen concentrations were related to higher COVID-19 infection rates, proposing that “pollen exposure weakens the immunity … by diminishing the antiviral interferon response.” They speculate that the effect could be stronger for those with allergies. As allergic people may also be more likely to show symptoms that may be mistaken for those of COVID-19 (2), we explored whether allergic people are more prone to get a rapid antigen test. Moreover, we assessed whether test results were indeed more likely to be positive among those with allergies.

In six cross-sectional surveys conducted between March and May 2021 (3), nonprobabilistic samples comprising ∼1,000 participants each were recruited, matching the distribution of age × gender and federal state of Germany (*n* = 5,971). The participants were asked whether they were allergic, whether they were tested with a rapid test because of symptoms, and, if so, whether they were infected (as confirmed by a more reliable PCR test) or not. Excluding missing responses resulted in a final sample of *n* = 5,653. The participants were 18 to 74 y old (mean = 45.04; SD = 15.71); 2,800 were male and 2,853 female; 1,600 participants indicated an allergy, and 521 had been tested because of symptoms. During the survey period, the pollen concentration in Germany was above the critical threshold for eliciting symptoms in allergic people (https://pollenscience.eu/).

As Fig. 1 shows, allergic people were indeed more likely to get tested, but their test results were not more likely to be positive. Thus, while Damialis et al. provide a plausible biological explanation for the relation between airborne pollen concentrations and COVID-19 cases, it must be considered that high pollen concentrations can also increase the number of tests because people experience symptoms that could be mistaken for COVID-19. Similar bias may exist for other conditions with symptoms that resemble COVID-19. While the statistical power may be too small to detect the obviously small effect on actual test results, the data suggest that the effect on testing should not be neglected. Given that 28% of Germans are allergic (4) and that those people are 1.33 to 1.93 times more likely to get tested, this would, in times of a high concentration of airborne pollen, imply an overall increase in testing of roughly 9 to 26%. Increased testing may at least briefly increase the number of identified cases by decreasing the dark figure. It will be relevant to observe whether the relation identified by the authors is attenuated when testing strategies shift from testing mainly symptomatic to also testing asymptomatic people (5). Even when the overall effect on case numbers may be small, this mechanism should be taken into account as the original results may cause more apprehension in allergic people than necessary.

**Fig. 1.**
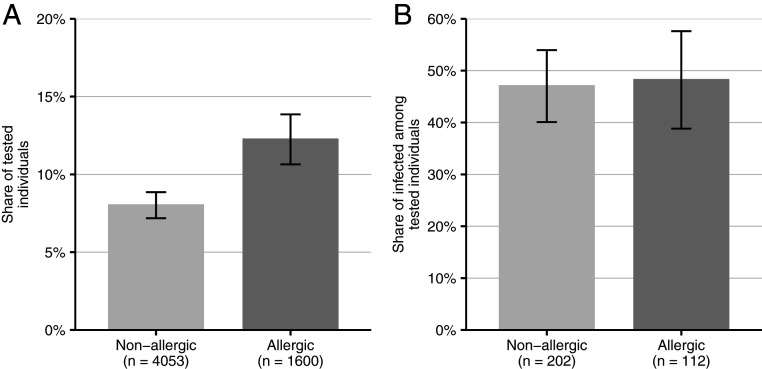
Differences in test use and infection rates between allergic and nonallergic people. Infection rates refer to PCR-confirmed infections in relation to all reliable test outcomes (negative rapid test results and PCR-confirmed positive rapid test results). Binary logistic regressions show that allergic participants were odds ratio (OR) = 1.60 (95% confidence interval [CI]: 1.33–1.93) times more likely to have been tested (*P* < 0.001; *A*) but not more likely to have a positive test result (OR = 1.05 [95% CI: 0.66–1.66], *P* = 0.84; *B*). The error bars visualize 95% CIs; “*n*” denotes the group size. The effect of being allergic on testing remains stable (OR = 1.61 [95% CI: 1.32–1.98], *P* < 0.001) when controlling for the local incidence rate (OR = 1.00 [95% CI: 1.00–1.00], *P* < 0.01, *n* = 5,022).

## Data Availability

Data, code, and details of the methods are available at Open Science Framework (https://osf.io/j5g7n/) (6).
